# A pilot study of therapeutic plasma exchange for serious SARS CoV-2 disease (COVID-19): A structured summary of a randomized controlled trial study protocol

**DOI:** 10.1186/s13063-020-04454-4

**Published:** 2020-06-08

**Authors:** Fahad Faqihi, Abdulrahman Alharthy, Mohammed Alodat, Daood Asad, Waleed Aletreby, Demetrios J. Kutsogiannis, Peter G. Brindley, Dimitrios Karakitsos

**Affiliations:** 1 Critical Care Department, King Saud Medical City, Riyadh, Kingdom of Saudi Arabia; 2 Critical Care Department, Al Imam Abdulrahman Al Feisal Hospital, Riyadh, Kingdom of Saudi Arabia; 3 Department of Critical Care, Faculty of Medicine and Dentistry, The University of Alberta, Edmonton, AB Canada; 4 Department of Internal Medicine, School of Medicine, South Carolina University, Columbia, SC USA; 5 Critical Care Department, Keck Medical School, USC, Los Angeles, CA USA

**Keywords:** COVID-19, Randomized controlled trial, Protocol, Therapeutic plasma exchange, Intensive care unit, Life-threatening COVID-19, Cytokine release syndrome, Acute respiratory distress syndrome, Multisystem organ failure

## Abstract

**Objectives:**

To evaluate the safety of therapeutic plasma exchange (TPE) in adult patients with serious/life-threatening COVID-19 requiring intensive care unit (ICU) admission, and associated 28-day mortality. Serious and life threatening COVID-19 are defined as per published literature (please, refer to the full protocol, Additional file 1). The rationale is that TPE can remove interleukins-3, 6, 8, 10, interferon-gamma and tumor necrosis factor-alpha. Thus, it may reduce the cytokine release syndrome associated with fulminant COVID-19 disease.

**Trial design:**

Pilot, interventional, open-label, randomized controlled multicenter trial.

**Participants:**

Inclusion criteria are: 1) age ≥ 18 years old; 2) intubation and intensive care unit (ICU) admission; 3) serious and/or life-threatening COVID-19 (please, refer to the full protocol, Additional file 1). SARS-CoV-2 infection is confirmed by Real-Time-Polymerase-Chain-Reaction (RT-PCR) assays using QuantiNova Probe RT-PCR kit (Qiagen) in a Light-Cycler 480 real-time PCR system (Roche, Basel, Switzerland).

Exclusion criteria are: 1) previous allergic reaction to plasma exchange or its ingredients (i.e., sodium citrate), 2) two consecutive negative RT-PCR tests for SARS-CoV-2 at least 24 hours apart, 3) mild COVID-19 not requiring ICU admission and 4) terminally ill patients receiving palliative care. The primary site will be King Saud Medical City (KSMC), Riyadh, Kingdom of Saudi Arabia (KSA). Also, the study will run in ICUs (Ministry of Health Cluster 1; Riyadh) and other centers in KSA pending their institutional review board (IRB) approval.

**Interventions and comparator:**

The intervention group will receive TPE, plus empiric treatment for COVID-19. TPE is administered using the Spectra Optia TM Apheresis System equipped with the Depuro D2000 Adsorption Cartridge (Terumo BCT Inc., USA). The first dose is 1.5 plasma volumes, followed by one plasma volume on alternate days or daily for five to seven total treatments. Spectra Optia TM Apheresis System operates with acid-citrate dextrose anticoagulant (ACDA) as per Kidney Disease Improving Global Outcomes (KDIGO) 2019 guidelines. Plasma is replaced with albumin 5% or fresh frozen plasma in patients with coagulopathy (prothrombin time >37 seconds; international normalized ratio >3; activated partial thromboplastin time >100 or fibrinogen level <100 mg/d). TPE sessions are performed daily over four hours and laboratory markers measured daily. The comparators are controls not receiving TPE but usual empiric treatment for COVID-19 as per institutional, national and international recommendations. Both groups will receive standard ICU supportive care.

**Main outcomes:**

Primary study end-point is 28-day mortality and safety of TPE in serious and/or life-threatening COVID-19. Safety will be evaluated by the documentation of any pertinent adverse and/or serious adverse effects related to TPE as per institutional, national and international (Food and Drug Administration) guidelines. Secondary outcomes are: i) improvement in Sequential Organ Function Assessment (SOFA) score ; ii) changes in inflammatory markers: serum C-reactive protein, lactate dehydrogenase, ferritin, d-dimers and interleukin-6; iii) days on mechanical ventilation and ICU length of stay.

**Randomization:**

Eligible consented patients are randomized (1:1 allocation) after stratification by ICU center and two PaO2/FIO2 ratio categories (> 150 and ≤ 150). Randomization occurs in variable block sizes of four to eight patients. A web-based randomization service, randomize.net, is used to allocate patients to their respective strata prior to the intervention or control therapy.

**Blinding (masking):**

Given the visibility of TPE machinery, the intervention will be unblinded; hence, no enrollment concealment will be expedited. The lack of allocation concealment will be mitigated by several measures (please, refer to the full protocol, Additional file 1).

**Numbers to be randomized (sample size):**

This pilot randomized trial aims to recruit a convenience sample of patients with serious and/or life-threatening COVID-19. Therefore, at least 20 patients are to be randomized to each group per participating center. We are hoping to consent and randomize approximately 60 patients in each group over a 3 to 6 months period giving a total of 120 participants.

**Trial Status:**

The protocol version 1 was approved 29/04/2020. Recruitment is ongoing, and began on 01/05/2020. We estimate completion by 29/10/2020.

**Trial registration:**

Registered at ISRCTN on 18/05/2020 (ISRCTN21363594; doi.10.1186/ ISRCTN21363594).

**Full protocol:**

The full protocol is attached as an additional file, accessible from the Trials website (Additional file 1). In the interest of expediting dissemination of this material, the familiar formatting has been eliminated; this letter serves as a summary of the key elements of the full protocol.

## Supplementary information

**Additional file 1.** Full study protocol.

## Data Availability

DK will have access to the final trial dataset. The dataset will be available from the corresponding author upon reasonable request. The contact e-mail address is karakitsosdimitrios@gmail.com

