# Supplementary material for: A pilot study of therapeutic plasma exchange for serious SARS CoV-2 disease (COVID-19): A structured summary of a randomized controlled trial study protocol
Source: Trials. 2020 Jun 8;21:506. doi: 10.1186/s13063-020-04454-4 (PMC7276972; doi:10.1186/s13063-020-04454-4)
Supplement: Supplementary file 1 — Additional file 1. Full study protocol. [file 13063_2020_4454_MOESM1_ESM.docx]

**
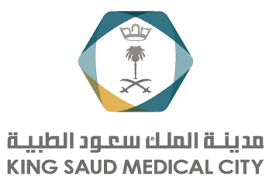

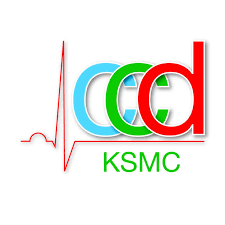
King Saud Medical City**

**Critical Care Department**

**A Pilot Randomized Clinical Trial of Therapeutic Plasma Exchange (TPE) in serious and/or life-threatening SARS-CoV-2 disease (COVID-19)**

**Institutional Review Board of King Saud Medical City, Riyadh, Kingdom of Saudi Arabia, protocol/serial number: H-01-R-053, IORG0010374, H1R1-29-Apr20-01**

**(ISRCTN21363594; doi.10.1186/ ISRCTN21363594)**

*Fahad Faqihi, Abdulrahman Alharthy,. Mohammed Alodat , Waleed Aletreby, Mohammed Alodat , Daood Saied Ahmad Dimitrios Karakitsos*

**Primary investigator**: Dr. Fahad Faqihi, *head of medica lCU* ,CCD,KSMC , Riyadh, Saudi Arabia. [faqihi@ksmc.med.sa](mailto:faqihi@ksmc.med.sa) **Project manager**: Dr. Dimitrios Karakitsos , ICU consultant , CCD,KSMC , Riyadh, Saudi Arabia.- karakitsosdimitrios@gmail.com **Co- investigators: Dr. Abdulrahman Alharthy**, ICU chairman, CCD,KSMC , Riyadh, Saudi Arabia.- araharthy@ksmc.med.sa **Dr. Mohammed Alodat** ,ICU consultant , CCD,KSMC , Riyadh, Saudi Arabia.- malodat@ksmc.med.sa **Dr. Waleed Aletreby**, associate consultant , CCD,KSMC , Riyadh, Saudi Arabia.- , CCD,KSMC , Riyadh, Saudi Arabia.- waleedaletreby@gmail.com **Mr. Daood Saied Ahmad Asad**, clinical nurse manager, CCD,KSMC , Riyadh, Saudi Arabia.- dasad@ksmc.med.sa

**Abstract**

Objectives: This study aims to evaluate the safety, and 28-day mortality, associated with administering therapeutic plasma exchange (TPE) to adult patients with serious/life-threatening COVID-19 requiring intensive care unit admission

Trial design: Randomized, open-label, controlled, multicenter study. Subjects who will meet the enrollment criteria stated below and provide informed consent will be randomly allocated to the intervention group (TPE) or control (no TPE). Both groups will receive empiric COVID-19 treatment and ICU supportive care.

**Introduction**

Coronaviruses (CoV) cause disorders that range from the common cold to severe illnesses such as the Middle East Respiratory Syndrome (MERS-CoV) and the Severe Acute Respiratory Syndrome (SARS-CoV, aka CoV-1). In December 2019, the onset of pneumonia of unknown origin occurred in Wuhan in Hubei Province, China. By the beginning of January 2020, this outbreak was confirmed to be caused by a novel coronavirus was confirmed, and was initially referred to a Severe Acute Respiratory Syndrome Corona-virus 2 related (so-called SARS-CoV-2). This has since been termed coronavirus disease 2019 (COVID-19) by the World Health Organization (WHO). High-throughput sequencing has revealed that SARS-CoV-2 is the seventh member of enveloped RNA coronavirus belonging to the Orthocoronavirinae subfamily. Despite COVID-19’s severity seeming lower than that of the two previous coronavirus diseases, i.e. SARS (severe acute respiratory syndrome) and MERS (Middle East respiratory syndrome), and contact transmission, and the relatively low mortality mean that it has spread worldwide and is now deemed a global pandemic. Clinical features of COVID-19 infection include fever, cough, respiratory symptoms such as shortness of breath, myalgias, headache, confusion, anosmia. Fulminant COVID-19 is usually associated with acute respiratory failure (ARF) and acute respiratory distress syndrome (ARDS), shock and multi-system organ failure (MSOF) as well as cytokine release syndrome (CRS). [1-8]

Fortunately most young patients and/or patients without comorbidities recover after a mild illness disease. In contrast, COVID-19 is more likely to be aggressive and fatal in elderly patients and those with comorbidities. Accordingly, death rates may reach up to 15%.[4-8] Laboratory detection of the SARS-CoV2 is based on detection of viral RNA by real-time reverse transcription- polymerase chain reaction (RT-PCR) with confirmation by nucleic acid sequencing when necessary. [1-8] GeneFinder™ COVID-19 Plus RealAmp Kit is the One-Step Reverse Transcription Real-Time PCR Kit used to detect the Novel Coronavirus (COVID-19) qualitatively through Reverse Transcription reaction and Real-Time Polymerase Chain Reaction. We will use this or equivalent method to confirm the diagnosis and will repeat the RT-PCR to make sure it has become negative in recovered patients. Currently, there is no definitive antiviral and/or other treatment for COVID-19 that has been validated.

The concept of the suggested protocol was derived from our own preliminary clinical observations. In brief, therapeutic plasma exchange (TPE) has the potential to be a rescue therapy in intubated ICU patients with fulminant COVID-19. This is because patients may present with ARDS, septic shock and/or MSOF and TPE has been used in severe sepsis, MSOF and fulminant SARS-CoV. Recently, the US and European Food and Drug Administration (FDA) has extended the use of TPE to include serious COVID-19 cases. The rationale is that TPE can remove interleukins-3, 6, 8, 10, interferon-gamma and tumor necrosis factor-alpha. Thus, it may reduce the cytokine storm and cascade associated with fulminant COVID-19 disease. In that sense, TPE can be a safe, easy and less complex alternative rescue therapy, especially when compared to other rescues treatment such as convalescent plasma transfusions, which requires the utilization of more resources and specialized equipment. [9-25]

In the Kingdom of Saudi Arabia, there are currently more than 60.000 COVID-19 cases and the death toll is increasing. This has led to the need for ICU care, despite the Kingdom’s prompt actions to apply lockdowns and curfew orders early on. COVID-19 is highly transmittable and is threatening our global health, economy and way of life. Accordingly, the development of effective therapeutic strategies to manage patients who have COVID-19 that is severe enough to requiring hospitalization, supplemental oxygen therapy and/or ICU admission is a research priority.

**Therapeutic plasma exchange logistics**

TPE is an elegant and readily available choice for treating serious COVID-19 cases. The technological and logistical background is currently available within most of MOH health care facilities. Our ICU at King Saud Medical City (KSMC) has been running a dedicated TPE program for the last 6 years. Hence, there is adequate clinical experience and dedicated staff for this therapy. The procedure is extremely simple and it will be explained in detail further down the text of this proposal.

**Research aim:**

This study aims to evaluate the safety, and 28-day mortality, associated with administering therapeutic plasma exchange (TPE) to adult patients with serious/life-threatening COVID-19 requiring intensive care unit admission

**Therapeutic options for COVID-19**

Currently, the management of COVID-19 has mainly focused on infection prevention, case detection, monitoring, and supportive care. Although there are reports on the potential efficacy of new therapeutic agents such as remdesevir and other antivirals but no specific antiviral treatment is recommended due to the absence of large prospective randomized-controlled studies. Therefore, it might be worthwhile to test the safety and efficacy of TPE in serious COVID-19 cases as we are already applying the therapy empirically for life saving purposes.

**Diagnostic considerations**

In those patients who have passed the viremia phase is highly desirable for a late diagnosis and also to ensure presence of antibodies that can help in fighting COVID-19. Recently, the Saudi FDA has approved a highly needed rapid test kit made by BIOZEK company (Inzek B.V. Vissenstraat 327324 AL, Apeldoorn The Netherlands) that detects qualitatively IgG and IgM Antibodies against SARS-CoV2 from whole blood, serum or plasma using a single-use cassette. This kit utilizes lateral flow chromatographic immunoassay and can produce results within 10 minutes only. The combination use of IgM and IgG test can reflect virus infection and the immune status of the body effectively.

Combination of real-time-polymerase-chain-reaction (RT-PCR) and antibody testing could render the diagnosis more robust and allow for detection of window and transmissibility timelines as well as facilitate studies about reinfection and natural immunity post-therapeutic interventions. Otherwise RT-PCR assays assays using QuantiNova Probe RT-PCR kit (Qiagen) in a Light-Cycler 480 real-time PCR system (Roche, Basel, Switzerland) will be utilized for detection of the virus in this study.

**Methodology**

This will be a multicenter pilot prospective clinical study evaluating the potential efficacy of TPE recruiting convenience sample of patients with severe COVID-19 who may present with ARF/ARDS, sepsis, MSOF and CRS. The primary site will be King Saud Medical City (KSMC), Riyadh, Kingdom of Saudi Arabia (KSA). Also, the study will run in ICUs (MOH Health Cluster 1; Riyadh) and other centers in KSA pending their IRB approval.

**Timeline**

The recruitment phase will extend over a period of 6 months aiming on including at least 20 patients in the study group (TPE) and 20 controls (patients with severe COVID-19 who will undergo standard therapy per MOH, KSA protocol and who will provide consent only to share their clinical and laboratory data) per participating center. Starting date: 10-05-20.

**Inclusion criteria**

We will use the confirmed case definition of SARS-CoV-2 infection (COVID-19) with POSITIVE RT PCR test for SARS-CoV-2 “using one of the SFDA approved kit used in KSA” as per current MOH / SCDPC (Waqayah) guidelines (derived from WHO and CDC ) [1, 2] see below.

1. Age ≥18 years old

2. Patient with COVID 19 confirmed as per case definition of CDC or MOH/Waqayah 3. 3. Patient requiring ICU admission due to severe COVID-19, life threatening disease and CRS

**Serious COVID-19 is defined as:**

Dyspnea

Altered level of consciousness

Respiratory frequency ≥ 30/min

Blood oxygen saturation ≤ 93% (despite oxygen therapy via nasal cannula 10L/min)

Partial pressure of arterial oxygen to fraction of inspired oxygen ratio < 300 (P/F ratio<300), and/or development of bilateral pulmonary infiltrates within 24 to 48 hours. [4-8]

**Life-threatening disease is defined as:**

ARDS defined according to the Berlin criteria and an Acute Physiology and Chronic Health Evaluation II (APACHE II) score ≥ 20

Sepsis and septic shock: severe sepsis is defined as life-threatening organ dysfunction caused by a dysregulated host response to infection, and septic shock as a subset of sepsis in which underlying circulatory and cellular metabolism abnormalities are profound enough to substantially increase mortality, with the clinical criteria of sepsis with persisting hypotension requiring vasopressors to maintain MAP ≥65 mm Hg and having a serum lactate level >2 mmol/L (18mg/dL) despite adequate volume resuscitation Multi-system organ failure (MSOF): defined as altered organ function in a critically ill patient requiring medical intervention to achieve homeostasis.

Development of cytokine release syndrome (CRS). [4-8]

**Criteria for developing CRS**

One or more of the following criteria present*

C-reactive protein > 100 or > 50 mg/L but doubled in the past 48 hours

Lymphocyte count < 0.6 x 109/L

Serum Interleukin-6 (IL-6) ≥ 3x upper normal limit

Ferritin > 300 ug/L (or surrogate) with doubling within 24 hours

Ferritin > 600 ug/L at presentation and Lactate dehydrogenase >250 U/L

Elevated D-dimer (> 1 mcg/mL)

*We defined low risk for developing CRS as the presence of 1 criterion; moderate risk if two to three criteria; high risk if ≥ 3 criteria*

**Exclusion criteria**

Negative RT-PCR test for SARS-CoV-2 Mild symptoms not requiring ICU admission

Terminally ill patient who are receiving palliative care History of allergy to TPE and its products

**TPE mode and therapeutic considerations**

A dose of 1.5 plasma volume exchanges will be used for the first dose then 1 plasma volume exchange on alternate days or daily for a total of 5-7 doses per individual clinical case scenario. Spectra Optia ^TM^ Apheresis System operates with acid-citrate dextrose anticoagulant (ACDA) as per Kidney Disease Improving Global Outcomes (KDIGO) 2019 guidelines. The plasma will be replaced with albumin 5% or fresh frozen plasma (in patients with coagulopathy). It is well documented that TPE removes various medications i.e., monoclonal autoantibodies; hence, use of Tocilizumab, steroids or other drugs as co-interventions will be not applied in our study group receiving TPE. The latter will not remove IgM molecules as they are too large. It appears that the therapy is more focused on controlling the inflammatory response by removing cytokines as per US FDA recommendations. [22]

**TPE technical details and infrastructure** TPE will be initiated using the Spectra Optia ^TM^ Apheresis System equipped with the Depuro D2000 Adsorption Cartridge (Terumo BCT Inc., USA). Sessions will be performed per US FDA recommendations for 4 hours per day to discreetly reduce the cytokine cascade. The number of sessions will be individualized according to pertinent clinical and laboratory parameters (i.e., improvement of P/F ratio, reduction of ferritin, IL-6 and LDH levels). Current US FDA recommendations state that: 1. SARS-CoV-2, the virus that causes COVID-19, can cause a serious or life-threatening disease or condition, including severe respiratory illness, to humans infected by this virus;

2. Based on the totality of scientific evidence available to FDA, it is reasonable to believe that the Spectra Optia Apheresis System with the Depuro D2000 Adsorption Cartridge may be effective in treating patients 18 years of age or older with confirmed COVID-19 admitted to the ICU with confirmed or imminent respiratory failure, and that the known and potential benefits of the Spectra Optia Apheresis System with the Depuro D2000 Adsorption Cartridge, when used to treat COVID-19 patients 18 years of age or older, outweigh the known and potential risks of the Spectra Optia Apheresis System with the Depuro D2000 Adsorption Cartridge; and

3. There is no adequate, approved, and available alternative to the emergency use of the Spectra Optia Apheresis System with the Depuro D2000 Adsorption Cartridge for the treatment of these COVID-19 patients.

The Spectra Optia Apheresis System with the Depuro D2000 Adsorption Cartridge devices mechanisms of function are as follows:

The D2000 Adsorption Cartridge operates in conjunction with the Terumo Spectra Optia device. The D2000 Adsorption Cartridge is integrated into the extracorporeal circuit, downstream from where the plasma is separated, into the Secondary Plasma Device (SPD) position of the circuit. After priming the D2000 Adsorption Cartridge and assembling the inlet and outlet lines to the plasma circuit, plasma filtration can be run for up to 4 hours, to be repeated as needed. The D2000 Adsorption Cartridge contains activated uncoated coconut shell (carbon granules) charcoal (100 gm), and the nonionic resins Amberlite XAD-7HP and Amberchrom GC300C. These adsorption materials have been demonstrated to be efficacious in the removal of statistically significant proportions of IL-3, IFN-gamma, IL-10, IL-1B, IL-6, IL-8, MCP-1, and TNF-alpha when compared to control. The adsorbents attract solutes through a variety of forces, including hydrophobic interactions, ionic (or electrostatic) attraction, hydrogen bonding, and van der Waals interactions. Management of the CRS associated with COVID-19 whilst treating the underlying pathogenesis may decrease patient morbidity. The reduction of cytokines must be done in a discrete, controlled fashion, to balance the patient’s immune response to the infection with the removal of the excess inflammatory cascade. Therefore, therapy with the D2000 will be administered for up to 4 hours per day. The Spectra Optia® Apheresis System Equipment (61000) is a non-patient, non-fluid contacting device. The following device settings have been validated for operation of the D2000 Adsorption Cartridge with the Terumo Spectra Optia with SPD software:

Prime divert volume: 200 mL

Notification pressure limit: 200 mmHg

Maximum pressure limit: 300 mmHg

Maximum plasma flow rate: 50 mL/min

Prime can be performed at up to 100 mL/min. At the start of treatment, the plasma pump flow rate may be adjusted up to 25 mL/min for the first 250 mL of plasma processed. Then, the plasma flow rate may be increased up to 50 mL/min as needed. This may be managed by the physician to operate between 15 and 50 mL/min, maintaining a pressure drop over the D2000 of less than 300 mm Hg. [22]

**Consent for TPE**

**Informed consent will be obtained in Arabic and English for this study.**

After obtaining informed consent, eligible patients who meet the inclusion criteria will undergo TPE sessions. As mentioned above the number of sessions will be individualized per clinical case scenario. Our preliminary experience is that patients with severe COVID-19 may require up to 5-7 sessions to improve.

Other supportive and therapeutic measures should continue according to the Saudi MOH approved protocols (Saudi Ministry of Health. Coronavirus Diseases 19 (COVID-19) guidelines. March 2020; version 1.2; (https://covid19.moh.gov.sa).

**Study design**

We are aiming on a pilot prospective design of 6 months duration. We are aiming to include at least 20 adult patients with severe COVID-19 as per inclusion criteria in our study group (TPE arm) per participating center. At least 20 COVID-19 patients and/or their next of Kin who will only consent for sharing their clinical and laboratory data (they will be treated per standard MOH protocol) will serve as a control group to compare the efficacy of the intervention per participating center. Our primary specific aim of this pilot randomized controlled trial is to evaluate the safety of utilizing TPE in critically ill COVID-19 patients and 28 day mortality. At present our best estimate of this critically ill population's mortality is 50%. As COVID-19 is a novel virus and the global pandemic interventions will result in dynamic changes in incident rates of this infection, we will be enrolling a convenience of critically ill COVID-19 patients in order to inform us of an estimated mortality rate and variability of organ dysfunction in order to inform the sample size calculation of a definitive randomized controlled trial in the future. Consequently we are hoping to consent and randomize approximately 60 patients in each group over a 3 to 6 months period. The two groups will be receiving the same standard mechanical ventilation protocols (i.e., proning, ventilation strategies) as per hospital protocol. Antibiotics, antivirals, other medications and dialysis/CRRT will be applied between groups as per hospital and Saudi MOH protocols for treatment of patients with serious COVID-19 (Saudi Ministry of Health. Coronavirus Diseases 19 (COVID-19) guidelines. March 2020; version 1.2; (https://covid19.moh.gov.sa).

**Study endpoint and outcome measures**

**Primary endpoints:**

28 day mortality and safety of TPE

**Secondary endpoints:**

Improvement of organ function as evaluated per SOFA score

Changes in laboratory inflammation markers

ICU length of stay

Number of days on mechanical ventilation

**Randomization** Eligible consented patients will be randomized after their stratification by ICU center and two PaO2/FIO2 ratio categories (> 150 and ≤ 150). Randomization will occur in variable block sizes of 4 to 8 patients. We will utilize a web-based randomization service, [randomize.net](http://randomize.net/) to allocate patients to their respective strata prior to the intervention or control therapy. Given the nature of the TPE technology, the intervention will be unblinded (open label); hence, no enrollment concealment will be expedited. However, the lack of allocation concealment will be mitigated as much as possible by the following measures. First, the primary outcome of mortality will not be disputable. Second, we have standardized co-interventions that impact on mortality as much as possible. These interventions include a) concomitant drug therapies for COVID-19, b) algorithms for mechanical ventilation including the early use of prone-ventilation, low tidal volume ventilation and neuromuscular blockade, c) the standard use of a broad spectrum antibiotic(s) for a minimum of 7 days to treat any potential superinfection be a bacterial pneumonia and d) standard criteria to initiate intermittent hemodialysis or CRRT such as hyperkalemia, profound metabolic acidosis, oliguria unresponsive to furosemide challenges of 1-1.5 mg/kg, e) the presence or development of coagulopathy defined as INR > 3.0 or Fibrinogen < 1.0 will be treated in a standard fashion in both the intervention and control group. We will define the date/time of appropriateness for ICU transfer irrespective of treatment allocation and recognizing that some COVID-19 patients randomized to the intervention group may improve to the point of appropriateness to transfer to the medical ward but requiring ICU care only to deliver remaining TPE doses as outlined in the protocol.

**Statistical analysis**

Continuous data will be summarized as mean ± standard deviation, and compared among groups with student t test or Wilxocon’s rank sum test as appropriate. Discrete data will be summarized as number (%) and compared by chi square or Fisher’s exact test as appropriate. All statistical tests are two tailed, considered significant with p value < 0.05 Survival analysis will be visually presented by Kaplan Meier’s Curve, and compared by hazard ratio. Statistical software SPSS 24.0 will be used.

Crude 30 and 90-days survival distributions between treatment [TPE] and control [no TPE] groups will be described using proportions and Kaplan Meier distributions. Crude differences will be tested using Chi Square test [for proportions] and a log-rank test [for the Kaplan Meier survival distributions]. A multivariable analysis of the primary 30 and 90-days survival outcome will be conducted using a Cox Proportional Hazard model where the main effect of treatment [TPE versus no TPE] and relevant confounding variables: age, gender, admission PaO2/FIO2 ratio, admission APACHE II and SOFA scores, presence of acquired coagulopathy and high vs low CRS group (already defined in previous paragraphs) will be entered into the model a-priori.

Similarly, a logistic regression multivariable model including the aforementioned variables will be fit to determine the independent effect of the TPE treatment on the binary outcome of 30 and 90 days survival.

Daily whole blood and effluent plasma samples will be obtained and assayed for various inflammatory markers IL-6, C-RP, ferritin etc. General estimating equations using random effects models will be used to describe the temporal changes in whole blood [in both treatment and control groups] and effluent plasma [in TPE group only]. Standard comparisons of the change in inflammatory markers using robust variance estimators will be used to compare the changes in these markers between treatment and control group.

**Data collection**

Clinical information of all enrolled cases will be retrieved from the hospital electronic/paper records system, including the: baseline demographic data, days of illness duration, presenting symptoms, radiological findings (CXR and Chest CT scans when possible), Sequential Organ Function Assessment (SOFA) and APACHE II scores. Data will be collected upon hospital/ICU admission, and prior to the first TPE session (Day 0) then on days 3, 7, 14, 30 accordingly and/or upon ICU/hospital discharge. Laboratory infectious marker testing results like culture from respiratory, urinary or blood culture will be collected accordingly.

Common laboratory and inflammatory markers (see below) will be collected on a daily basis:

1. CBC differential to include percent and absolute lymphocyte count and percent and absolute neutrophil count

2. Chemistry panel to include Total protein, Albumin, Lactate Dehydrogenase (LDH), Aminotransferases (ALT, AST), Procalcitonin

3. Cardiac biomarkers (e.g. cardiac troponins)

4. CK

5. Ferritin, LDH

6. Full coagulation profile to include PT, APTT, fibrinogen and D-dimer levels

7. C Reactive Protein (CRP) and erythrocyte sedimentation rate (ESR)

8. Interleukin-6

RT-PCR test for SARS-CoV-2 will be done and will be monitored during treatment and at day 14 of recovery or discharge, whatever is later. Also, we will be monitoring: mechanical ventilation (MV) and modes, intranasal oxygen inhalation, non-invasive mechanical ventilation, number of days of MV or nasal oxygen support, medication regimens (i.e., hydroxychloroquine, anti-viral therapies, steroids, tocilizumab, etc.), complications (including acute renal failure, acute coronary syndrome, myocarditis, acute respiratory distress syndrome, GI complications, CNS complications and nosocomial infections).

**Response assessment**

Daily clinical assessment by physician:

Vital signs including temperature, blood pressure, respiratory rate, heart rate. Oxygen saturation, arterial blood gases, and P/F ratios.

Ventilator requirement and the modes employed. Vasopressors requirement, serum lactate levels.

Complete blood counts, liver function tests, urea, creatinine, inflammation markers and electrolytes daily. Glasgow coma scale.

Fluid balance.

X-ray / CT changes.

Organs functions assessment by SOFA score.

TPE doses and frequency requirement.

SARS-CoV-2 RNA will be tested on recovery (or deterioration to determine alternative etiology).

**Monitoring and Safety**

Periodic monthly report will be generated for IRB monitoring. TPE is a routine practice in health care facilities. All known adverse events (AE) and serious adverse events (SAE) will be collected as per the SFDA, CBAHI and AABB reporting standard. SAE will be reported by the study team to the IRB chairman and PI within 12 hours of the event.

**Expected Outcome**

TPE is a cheap modality and readily available. It is expected to save lives and improve the clinical, laboratory and radiological features of at least 60% of the patients severely affected by COVID-19.

**Outcome Utilization**

Decrease in morbidity and mortality of severe COVID-19 disease cost-effectively, leading to improved quality of healthcare in Saudi Arabia.

**Value to the Kingdom Vision 2030**

Self-sufficiency in the treatment of serious diseases affecting the masses in a cost-effective manner is among the top priorities in Vision 2030.

| **References** [1]. Guan WJ, Ni ZY, Hu Y, et al. Clinical Characteristics of Coronavirus Disease 2019 in China. China Medical Treatment Expert Group for Covid-19. N Engl J Med 2020; Feb 28. [2]. Zhou F, Yu T, Du R, et al. Clinical course and risk factors for mortality of adult inpatients with COVID-19 in Wuhan, China: a retrospective cohort study. Lancet 2020; 11 March. [3]. Grasselli G, Zangrillo A, Zanella A, et al. Baseline Characteristics and Outcomes of 1591 Patients Infected With SARS-CoV-2 Admitted to ICUs of the Lombardy Region, Italy. JAMA 2020; 6 April [4]. Duan K, Liu B, Li C. et al. Effectiveness of convalescent plasma therapy in severe COVID-19 patients. Proc Natl Acad Sci U S A. 2020; Apr 6. [5]. Shen C, Wang Z, Zhao F, et al. Treatment of 5 Critically Ill Patients With COVID-19 With Convalescent Plasma. JAMA 2020; 27 March. [6]. Zhao R, Li M, Song H, et al., Early detection of SARS-CoV-2 antibodies in COVID-19 patients as a serologic marker of infection. Clin Infect Dis 2020; May 1. [7]. Deng Y, Liu W, Liu K, et al. Clinical characteristics of fatal and recovered cases of coronavirus disease 2019 (COVID-19) in Wuhan, China: a retrospective study. Chin Med J 2020; March 2020. [8]. Saudi Ministry of Health. Coronavirus Diseases 19 (COVID-19) guidelines. March 2020; version 1.2; (<https://covid19.moh.gov.sa>). [9]. Bellani G, Laffey JG, Pham T, et al. Epidemiology, Patterns of Care, and Mortality for Patients With Acute Respiratory Distress Syndrome in Intensive Care Units in 50 Countries. JAMA. 2016; 23;315(8):788-800. [10]. Ferguson ND, Fan E, Camporota L et al. The Berlin definition of ARDS: an expanded rationale, justification, and supplementary material. Intensive Care Med 2012; 38(10):1573-82. [11]. Salluh JI, Soares M. ICU severity of illness scores: APACHE, SAPS and MPM. Curr Opin Crit Care 2014; 20(5):557-65. [12]. Rhodes A, Evans LE, Alhazzani W et al. Surviving Sepsis Campaign: International Guidelines for Management of Sepsis and Septic Shock: 2016 Intensive Care Med 2017; 43(3):304-377. [13]. Levy MM, Evans LE, Rhodes A. The Surviving Sepsis Campaign Bundle: 2018 update. Intensive Care Med 2018; 44(6):925-928. [14]. World Health Organization, Clinical management of severe acute respiratory infection when Novel coronavirus (nCoV) infection is suspected: Interim guidance. https://www.who.int/ publications-detail/clinical-management-of-severe-acute-respiratory-infectionwhen- novel-coronavirus-(ncov)-infection-is-suspected. [15]. <https://covid19.cdc.gov.sa>. [16]. Nalla AK, Casto AM, HuangMW, et al. Comparative performance of SARS-CoV-2 detection assays using seven different primer/probe sets and one assay kit. J Clin Microbiol 2020; April 8. [17]. Wölfel R, Corman VM, GuggemosW, et al. Virological assessment of hospitalized patients with COVID-2019. Nature 2020; April 1. [18]. WangW, Xu Y, Gao R, et al. Detection of SARS-CoV-2 in different types of clinical specimens. JAMA 2020; March 11. [19]. Liu R, Han H, Liu F, et al. Positive rate of RT-PCR detection of SARS-CoV-2 infection in 4880 cases from one hospital in Wuhan, China, from Jan to Feb 2020. Clin Chim Acta 2020; 505:172-175. [20]. Chan JF, Yip CC, To KK, et al. Improved Molecular Diagnosis of COVID-19 by the Novel, Highly Sensitive and Specific COVID-19-RdRp/Hel Real-Time Reverse Transcription-PCR Assay Validated In Vitro and with Clinical Specimens. J Clin Microbiol 2020; Apr 23;58(5). [21]. Corman VM, Landt O, Kaiser M, et al. Detection of 2019 novel coronavirus (2019-nCoV) by real-time RT-PCR. Euro Surveill 2020; 25(3):pii2000045. [22]. https://www.fda.gov/media/136837/download: D2000 Cartridge Operation Manual for Use of D2000 with the Terumo Spectra Optia™ Apheresis System; for use in the U.S. under FDA EUA200148: Authorization for Emergency Use in patients with COVID-19 admitted to the ICU with confirmed or imminent respiratory failure. [23]. Wang AY, Akizawa T, Bavanandan S, et al. 2017 Kidney Disease: Improving Global Outcomes (KDIGO) Chronic Kidney Disease-Mineral and Bone Disorder (CKD-MBD) Guideline Update Implementation: Asia Summit Conference Report. Kidney Int Rep 2019; 4(11):1523-1537. [24]. Pai M, Moffat KA, Plumhoff E, Hayward CPM. Critical Values in the Coagulation Laboratory: Results of a Survey of the North American Specialized Coagulation Laboratory Association. American Journal of Clinical Pathology 2011; 136 (6): 836–841. [25]. Seymour CW, Liu VX, Iwashyna TJ. Assessment of Clinical Criteria for Sepsis: For the Third International Consensus Definitions for Sepsis and Septic Shock (Sepsis-3). JAMA 2016; Feb 23;315(8):762-74. |
| --- |
